# Complete Coding Sequences of 23 South African Domestic and Wildlife Rabies Viruses

**DOI:** 10.1128/MRA.00621-20

**Published:** 2020-09-17

**Authors:** Claude Sabeta, Debrah Mohale, Baby Phahladira, Ernest Ngoepe, Antoinette Van Schalkwyk, Kgaogelo Mogano, George Chirima, Toru Suzuki, Kohei Makita

**Affiliations:** a Agricultural Research Council—Onderstepoort Veterinary Institute (ARC-OVI), Onderstepoort, Pretoria, South Africa; b University of Pretoria, Veterinary Tropical Diseases Department, Onderstepoort, Pretoria, South Africa; c Agricultural Research Council, Soil Climate and Water, Pretoria, South Africa; d Centre for Geoinformation Science, Department of Geography, Geoinformatics and Meteorology, University of Pretoria, Pretoria, South Africa; e Rakuno Gakuen University, Sapporo, Hokkaido, Japan; Portland State University

## Abstract

South African rabies viruses originating from dogs and jackals (canid viruses) are closely related and highlight cross-species transmission events between the two canine species. Rabies due to the canid lyssavirus variant is a significant public health matter in this country. The complete coding sequences of 23 canid lyssaviruses from South Africa are reported here.

## ANNOUNCEMENT

Rabies virus (RABV) is the prototype species of the *Lyssavirus* genus (family *Rhabdoviridae*, order *Mononegavirales*) (1) and causes encephalitis in all warm-blooded vertebrates. In South Africa, RABV infects both domestic and wild carnivore species (2). The domestic dog is the main vector species responsible for the transmission of rabies to humans (3), resulting in at least 59,000 human deaths annually, and the majority of these (≥95%) are recorded in Africa and Asia (2).

Specimens were collected by state veterinarians from animals in northern South Africa (Limpopo and Mpumalanga provinces) showing typical signs of central nervous system infection, preserved in glycerol-saline, and transported to the laboratory (see Table 1 for epidemiological information on the samples). The composite brain tissues were then subjected to a direct fluorescent antibody test for lyssavirus antigen (4). Total viral RNAs were extracted from the original lyssavirus-infected brain tissues and prepared for next-generation sequencing (NGS) using the HiSeq (Illumina, San Diego, CA, USA) platform. Briefly, the TRIzol-extracted viral RNA was depleted of host genomic DNA and rRNA using the on-column DNase treatment in the RNeasy Plus kit (Qiagen) and Terminator 5′-phosphate-dependent exonuclease (Epicentre Biotechnologies), as described previously (5–7). Double-stranded (ds) cDNA was synthesized from 50 ng RNA using a random cDNA synthesis system kit (Roche, Basel, Switzerland) according to the manufacturer’s instructions. The ds cDNA was purified using AMPure XP magnetic beads (Beckman Coulter, Brea, CA, USA), and 1 ng was used for the Nextera XT DNA sample preparation kit (Illumina). A sequencing library was prepared for each sample according to the manufacturer’s instructions and sequenced on an Illumina HiSeq with 2 × 125-bp paired-end reads following standard Illumina protocols, resulting in between 0.002 and 5.6 Gb data per isolate (Table 1). For each of the isolates, contigs were generated through both *de novo* assembling of all the reads and mapping of the same reads to existing rabies genomes (GenBank accession numbers KT336433 to KT336436) using default settings in CLC Genomics Workbench v.9 (Qiagen). A single consensus sequence was produced for each sample from an assembly of all the contigs previously generated. The newly obtained consensus sequence was used as a template to map all the original reads in order to estimate the average coverage and possible variants (see Table 1). The genetic organization of the full genomes of the South African RABVs was consistent with that of other previously characterized lyssaviruses (5–7). The sequence length and percent G+C content of each of the samples are listed in Table 1. Each sequence contains the complete coding regions of the nucleoprotein (N), glycoprotein (G), matrix protein (M), phosphoprotein (P), and the RNA-dependent polymerase (L) found in rabies viruses. Genetic analysis of these canid RABVs demonstrated that they are of the Africa 1-b lineage and have a high degree of sequence similarity (≥96.5%) (mean distance, 0.023; standard error [SE], 0.001), irrespective of their host species and locality of origin (1, 7–9).

**TABLE 1:** Epidemiological information of the canine viruses sequenced in this study

Virus no.	Lab ref no.^*a*^	Yr of submission	Species from which sample originated	Size of raw data (GB)	No. (%) of reads mapped to new consensus sequence	Sequence length (bp)	% G+C content	BioProject accession no.	GenBank accession no.
1	125/15	2015	Canis familiaris	2.62	159,506 (0.76)	11,923	45.4	SRR12012256	MT454631
2	155/15	2015	Proteles cristatus	2.30	7,365 (0.04)	11,902	45.5	SRR12012255	MT454632
3	361/15	2015	*Proteles cristatus*	2.73	4,365 (0.02)	11,908	45.6	SRR12012244	MT454633
4	471/15	2015	*Canis familiaris*	2.65	72,025 (0.34)	11,923	45.4	SRR12012240	MT454634
5	682/15	2015	*Canis familiaris*	5.63	31,529 (0.07)	11,923	45.3	SRR12012239	MT454635
6	516/16	2016	*Canis familiaris*	1.73	217,555 (1.57)	11,923	45.3	SRR12012238	MT454636
7	583/16	2016	*Canis familiaris*	2.53	309,360 (1.53)	11,923	45.4	SRR12012237	MT454637
8	631/16	2016	Otocyon megalotis	4.16	6,656 (0.02)	11,923	45.4	SRR12012236	MT454638
9	635/16	2016	*Canis familiaris*	1.39	166,479 (1.5)	11,923	45.3	SRR12012235	MT454639
10	676/16	2016	*Otocyon megalotis*	3.53	5,640 (0.02)	11,923	45.4	SRR12012234	MT454640
11	690/16	2016	*Canis familiaris*	0.002	37.321 (0.22)	11,923	45.3	SRR12012254	MT454641
12	725/16	2016	*Proteles cristatus*	2.90	6,971 (0.03)	11,923	45.5	SRR12012253	MT454642
13	269/17	2017	*Canis familiaris*	4.40	218,019 (0.62)	11,922	45.6	SRR12012252	MT454643
14	400/17	2017	*Canis familiaris*	1.94	37,211 (0.24)	11,922	45.6	SRR12012251	MT454645
15	454/17	2017	Canis mesomelas	2.80	123,359 (0.55)	11,922	45.6	SRR12012250	MT454646
16	460/17	2017	*Canis mesomelas*	2.95	21,259 (0.09)	11,922	45.6	SRR12012249	MT454647
17	466/17	2017	*Canis mesomelas*	4.43	10,620 (0.03)	11,923	45.3	SRR12012248	MT454648
18	474/17	2017	*Canis mesomelas*	1.58	26,616 (0.21)	11,922	45.6	SRR12012247	MT454649
19	477/17	2017	*Otocyon megalotis*	2.14	92,643 (0.54)	11,922	45.6	SRR12012246	MT454650
20	480/17	2017	*Canis mesomelas*	2.90	53,270 (0.23)	11,922	45.6	SRR12012245	MT454651
21	483/17	2017	*Canis mesomelas*	2.40	7,664 (0.04)	11,923	45.3	SRR12012243	MT454652
22	502/17	2017	*Canis mesomelas*	2.26	81,394 (0.45)	11,923	45.3	SRR12012242	MT454653
23	503/17	2017	*Canis mesomelas*	2.21	70,808 (0.4)	11,923	45.3	SRR12012241	MT454654

a Sample submitted to Agricultural Research Council—Onderstepoort Veterinary Institute (ARC-OVI) for laboratory confirmation of RABV.

### Data availability.

The complete coding sequences were submitted to GenBank and are available under accession numbers MT454631 through MT454654 (Table 1) and BioProject accession number PRJNA638742.

## References

[1] Afonso CL, Amarasinghe GK, Bányai K, Bào Y, Basler CF, Bavari S, Bejerman N, Blasdell KR, Briand F-X, Briese T, Bukreyev A, Calisher CH, Chandran K, Chéng J, Clawson AN, Collins PL, Dietzgen RG, Dolnik O, Domier LL, Dürrwald R, Dye JM, Easton AJ, Ebihara H, Farkas SL, Freitas-Astúa J, Formenty P, Fouchier RAM, Fù Y, Ghedin E, Goodin MM, Hewson R, Horie M, Hyndman TH, Jiāng D, Kitajima EW, Kobinger GP, Kondo H, Kurath G, Lamb RA, Lenardon S, Leroy EM, Li C-X, Lin X-D, Liú L, Longdon B, Marton S, Maisner A, Mühlberger E, Netesov SV, Nowotny N, 2016 Taxonomy of the order Mononegavirales: update 2016. Arch Virol 161:2351–2360. doi:10.1007/s00705-016-2880-1. 27216929 PMC4947412

[2] Sabeta CT, Bingham J, Nel LH 2003 Molecular epidemiology of canid rabies in Zimbabwe and South Africa. Virus Res 91:203–211. doi:10.1016/s0168-1702(02)00272-1. 12573499

[3] Knobel DL, Cleaveland S, Coleman PG, Fevre EM, Meltzer MI, Miranda MEG, Shaw A, Zinsstag J, Meslin F-X 2005 Re-evaluating the burden of rabies in Africa and Asia. Bull World Health Organ 83:360–368. 15976877 PMC2626230

[4] Dean DJ, Abelseth MK, Atanasiu P 1996 The fluorescent antibody test, p 88–95. *In* Meslin F-X, Kaplan MM, Koprowski H (ed), Laboratory techniques in rabies, 4th ed World Health Organization, Geneva, Switzerland.

[5] Sabeta C, Phahladira B, Marston DA, Wise EL, Ellis RJ, Fooks AR 2015 Complete genome sequences of six southern African rabies viruses. Genome Announc 3:e01085-15. doi:10.1128/genomeA.01085-15. 26430028 PMC4591300

[6] Troupin C, Dacheux L, Tanguy M, Sabeta C, Blanc H, Bouchier C, Vignuzzi M, Duchene S, Holmes EC, Bourhy H 2016 Large-scale phylogenomic analysis reveals the complex evolutionary history of rabies virus in multiple carnivore hosts. PLoS Pathog 12:e1006041. doi:10.1371/journal.ppat.1006041. 27977811 PMC5158080

[7] Brunker K, Marston DA, Horton DL, Cleaveland S, Fooks AR, Kazwala R, Ngeleja C, Lembo T, Sambo M, Mtema ZJ, Sikana L, Wilkie G, Biek R, Hampson K 2015 Elucidating the phylodynamics of endemic rabies virus in eastern Africa using whole-genome sequencing. Virus Evol 1:vev011. doi:10.1093/ve/vev011. 27774283 PMC5014479

[8] Ngoepe EC, Fehlner-Gardiner C, Wandeler AI, Sabeta CT 2014 Antigenic characterisation of lyssaviruses in South Africa. Onderstepoort J Vet Res 81:a711. doi:10.4102/ojvr.v81i1.711. 25685866

[9] Nel LH, Thomson GR, Von Teichman BF 1993 Molecular epidemiology of rabies virus in South Africa. Onderstepoort J Vet Res 60:301–306. 7777315

